# mRNA-seq whole transcriptome profiling of fresh frozen versus archived fixed tissues

**DOI:** 10.1186/s12864-018-4761-3

**Published:** 2018-05-30

**Authors:** Noa Bossel Ben-Moshe, Shlomit Gilad, Gili Perry, Sima Benjamin, Nora Balint-Lahat, Anya Pavlovsky, Sharon Halperin, Barak Markus, Ady Yosepovich, Iris Barshack, Einav Nili Gal-Yam, Eytan Domany, Bella Kaufman, Maya Dadiani

**Affiliations:** 1 Department of Physics of Complex Systems, Weizmann Institute of Science, Rehovot, Israel; 2 The Nancy and Stephen Grand Israel National Center for Personalized Medicine, Weizmann Institute of Science, Rehovot, Israel; 3 Chaim Sheba Medical Center, Cancer Research Center, 5262100 Tel-Hashomer, Israel; 4 Chaim Sheba Medical Center, Institute of Pathology, Tel-Hashomer, Israel; 5 Chaim Sheba Medical Center, Institute of Oncology, Tel-Hashomer, Israel; 6 Sackler Faculty of Medicine, Tel Aviv University, Tel Aviv, Israel

**Keywords:** RNA sequencing, FFPE, Poly-a capturing, Ribosomal depletion, Breast cancer

## Abstract

**Background:**

The main bottleneck for genomic studies of tumors is the limited availability of fresh frozen (FF) samples collected from patients, coupled with comprehensive long-term clinical follow-up. This shortage could be alleviated by using existing large archives of routinely obtained and stored Formalin-Fixed Paraffin-Embedded (FFPE) tissues. However, since these samples are partially degraded, their RNA sequencing is technically challenging.

**Results:**

In an effort to establish a reliable and practical procedure, we compared three protocols for RNA sequencing using pairs of FF and FFPE samples, both taken from the same breast tumor. In contrast to previous studies, we compared the expression profiles obtained from the two matched sample types, using the same protocol for both. Three protocols were tested on low initial amounts of RNA, as little as 100 ng, to represent the possibly limited availability of clinical samples. For two of the three protocols tested, poly(A) selection (mRNA-seq) and ribosomal-depletion, the total gene expression profiles of matched FF and FFPE pairs were highly correlated. For both protocols, differential gene expression between two FFPE samples was in agreement with their matched FF samples. Notably, although expression levels of FFPE samples by mRNA-seq were mainly represented by the 3′-end of the transcript, they yielded very similar results to those obtained by ribosomal-depletion protocol, which produces uniform coverage across the transcript. Further, focusing on clinically relevant genes, we showed that the high correlation between expression levels persists at higher resolutions.

**Conclusions:**

Using the poly(A) protocol for FFPE exhibited, unexpectedly, similar efficiency to the ribosomal-depletion protocol, with the latter requiring much higher (2–3 fold) sequencing depth to compensate for the relative low fraction of reads mapped to the transcriptome. The results indicate that standard poly(A)-based RNA sequencing of archived FFPE samples is a reliable and cost-effective alternative for measuring mRNA-seq on FF samples. Expression profiling of FFPE samples by mRNA-seq can facilitate much needed extensive retrospective clinical genomic studies.

**Electronic supplementary material:**

The online version of this article (10.1186/s12864-018-4761-3) contains supplementary material, which is available to authorized users.

## Background

Gene expression profiling of tumor samples is a powerful technique for identifying prognostic and predictive biomarkers. To date, all large scale transcriptomic profiling of cancer were performed on frozen tissue samples and required international efforts; nevertheless, analysis of the results was limited by availability of the numbers of frozen tumor samples that were obtained from patients with long-term clinical follow up [1, 2]. On the other hand, there exist large diagnostic repositories of archived tissues, with matched clinical records on disease progression and outcome, which could potentially comprise invaluable resources for comprehensive genomic studies of cancer. Exploiting archived reservoirs could allow unprecedented large-scale retrospective investigations of longitudinal samples, focusing on specific subtypes or ethnic groups of interest, without the need for long-term prospective collection of samples. Such informative datasets could facilitate biomarker discovery and personalized drug development.

Archived tissues are preserved as formalin-fixed paraffin-embedded (FFPE) blocks, which is the standard preparation format for pathological diagnosis. The quality of FFPE-extracted RNA is highly variable due to fragmentation of RNA transcripts, chemical modifications and cross-linking of nucleic acids and proteins [3, 4] as well as variability in tissue handling and processing [5]. These factors are influenced by various methods for tissue fixation and by archiving time of tissue samples. Hence, due to the relative low quality of FFPE-derived RNA, applying standard protocols for whole transcriptome analysis is a challenging task.

In recent years the field has moved from microarray based profiling to RNA-sequencing (RNA-seq), an unbiased method which provides greater analytical depth and increased dynamic range for gene expression measurement [6, 7]. RNA-seq protocols commonly include steps for enrichment of exonic RNA sequences and removal of ribosomal RNA. The conventional protocol, known as mRNA-Seq, is based on capturing polyadenylated (poly(A)) RNA transcripts with oligo(dT) primers, thereby depleting the highly abundant ribosomal RNA (rRNA) and all other non-poly(A) fragments [8]. The concern in utilizing mRNA-Seq for FFPE samples is that degraded or modified poly(A) tail of transcripts will not perfectly anneal to oligo(dT) and the captured short transcripts will not comprise an unbiased, accurate representation of the transcriptome. Other protocols eliminate rRNA by capturing these highly abundant transcripts and removing them by either magnetic beads (i.e. RiboZero by Illumina) or by enzymatic digestion, (i.e., RNAse H or Ovation by NuGEN) [9–12].

Several previous studies evaluated the feasibility of RNA-seq to reliably profile gene expression comparing matched FF and FFPE samples. However, most studies used the gold-standard poly(A) mRNA-seq protocol for FF samples compared to ribosomal-depletion protocol for FFPE [10, 12–14], out of the assumption that mRNA-seq will not optimally capture degraded mRNAs. Such comparisons mix effects of the different sample types with those of the protocol used. In the current study we perform an unbiased evaluation of RNA-seq of archived tumor tissues by comparing the same library preparation methods for both FF and FFPE matched tumor samples and for small amounts of total RNA starting material. In addition to comparing the coverage and mapping parameters of FF and FFPE, we addressed the question of the reproducibility characteristics of the sample types and the methods, when trying to estimate differential gene expression, with particular focus on quantifying expression of clinically relevant genes, derived from the METABRIC dataset [2].

## Results

Three pairs of matched FF/FFPE tumor samples were collected, with a moderate archival time of about 4–5 years (T1-T3). To compare the efficiency of the various RNA-seq protocols we used the same library preparation protocol for both FF and FFPE samples. Three protocols were tested: illumina Truseq RNA after poly(A) selection (mRNA-seq); Truseq after ribosomal depletion (RiboZero); and Nugen Ovation with Ribosomal depletion. As sometimes archived biopsies are limited in size, we also compared 2 different amounts of starting material, 500 and 100 ng of RNA, to assess the lower starting material limitations of the protocols. In addition, to evaluate the limitation of the mRNA-seq method for highly degraded RNA, we analyzed additional 3 FFPE tumor samples archived for more than 10 years (with no matching FF samples; T4-T6). RNA integrity was high for the FF samples (RIN number 6–7) and low for the FFPE samples (1.8 or below detection limit) (see Additional file 1: Table S1).

### Transcriptome mapping and coverage

Sequencing reads were aligned to the human genome to assess the fraction of mapped transcripts. Percentage of uniquely mapped reads varied between the sample types in favor of the FF samples (Table 1 and Additional file 2: Table S2). Older FFPE samples exhibited lower fraction of aligned reads, probably due to lower RNA quality, however the fraction of exonic reads in the old samples was relatively high, so that the number of exonic reads sufficed for gene expression analysis (Table 1 and Additional file 2: Table S2). The fraction of unmapped reads obtained from the NuGEN Ovation for the FFPE samples protocol was very high (~ 60%), resulting in very low percentage of exonic reads out of the total number of reads (~ 4%); therefore, we excluded this protocol from further analysis (Table 1 and Additional file 2: Table S2). mRNA-seq and RiboZero exhibited comparable efficiency of rRNA removal for FFPE samples but showed high variance for the FF samples (1.3% versus 24.7%, see Table 1).

**Table 1:** Mapping percentages for the different RNA-seq methods and samples

	mRNA-seq			RiboZero		Nugen	
	FF	FFPE	FFPE old	FF	FFPE	FF	FFPE
	*n =* 6	*n =* 5	*n =* 3	*n =* 3	*n =* 3	*n =* 3	*n =* 6
Exons	58 (51–63)	29.2 (16.6–38.8)	20.1 (13.8–32.2)	21.4 (14.9–30)	8.4 (0.6–12.7)	20.3 (16–22.7)	3.9 (0.8–7)
Intronic/intergenic	25 (20–32)	28.2 (22.3–32.6)	23.3 (18.9–27.4)	44.4 (35.1–55.5)	70.2 (61.7–75.2)	65.1 (63.1–67.9)	32.5 (24.5–43.1)
rRNA	1.3 (0.9–1.6)	12.7 (4.17–17.8)	6.4 (2.7–12.9)	24.7 (1.15–45.3)	5.3 (0.9–13.4)	0.07 (0.05–0.08)	1.2 (0.3–2.2)
Multiple alignment	11.9 (9.5–15)	5.9 (3.8–7.2)	4.2 (2.63–6)	6.3 (2–10)	6.5 (6.2–6.6)	9.6 (8.7–10.3)	3.6 (2.7–4.7)
Unmapped	3.8 (3.7–4)	24 (10.9–44.9)	45.7 (31.6–54)	3 (2.7–3.3)	9.6 (5.3–17.8)	4.8 (4.2–6.1)	58.7 (43–67.8)

Values presented as mean percentage (range)

*n* = number of measurements (#of tumor samples X number of initial RNA amounts)

**Fig. 1:**
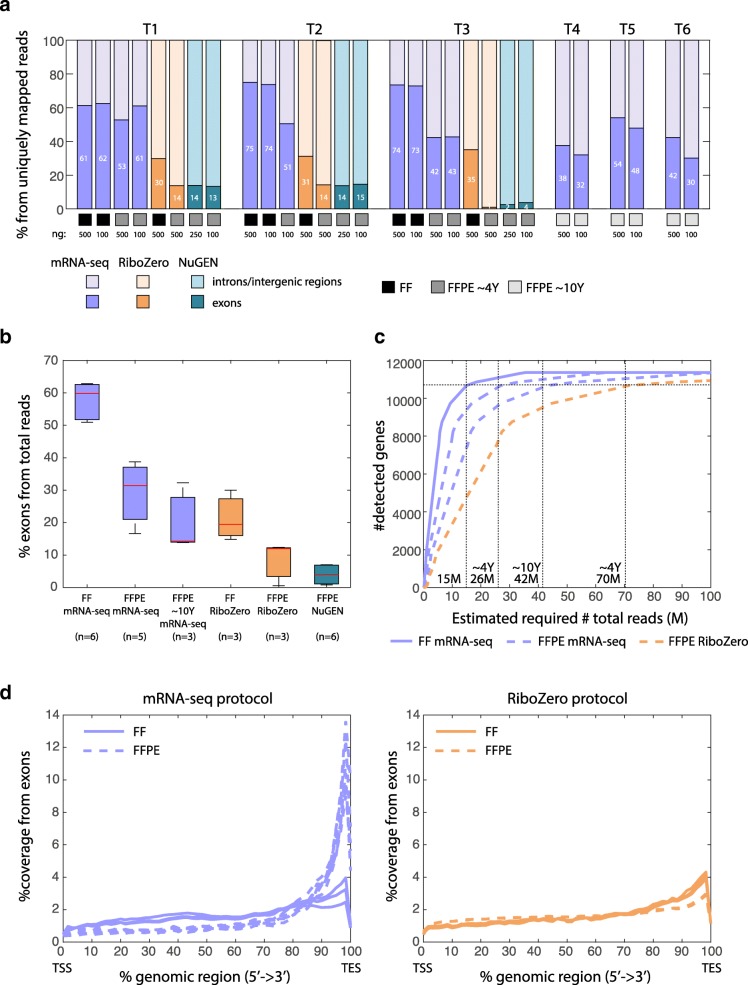
Mapping and coverage information. (**a**) The percentage of reads mapped to exons and to introns/intergenic regions out of the total number of uniquely mapped reads per sample. Color-code: purple for mRNA-seq protocol, orange for RiboZero protocol and blue for NuGEN ovation protocol. Black: FF samples (T1-T3), gray: ~ 4 years old FFPE samples (T1-T3), and light gray: ~ 10 years old FFPE samples (T4-T6, done only with mRNA-seq protocol). The amount of starting material, in ng, is indicated below the bars corresponding to each sample. (**b**) Box-plot of the percentages of reads uniquely mapped to exons out of total number of reads, for each sample type (FF mRNA-seq (T1-T3; *n* = 6); ~ 4 years old FFPE mRNA-seq (T1-T3; *n* = 5); ~ 10 years old FFPE mRNA-seq (T4-T6; *n* = 3); FF RiboZero (T1-T3; *n =* 3); FFPE RiboZero (T1-T3; *n =* 3); FFPE NuGEN ovation (T1-T3; *n =* 6)). *n =* number of measurements (#of tumor samples x number of initial RNA amounts). (**c**) The estimated number of detected genes as a function of the total number of reads for each sample type (FF mRNA-seq samples: purple solid line; FFPE mRNA-seq samples: purple dashed line, shown separately for ~ 4 and ~ 10 years old samples; FFPE RiboZero samples: orange dashed line). The horizontal dashed line represents the estimated coverage required for each sample type to get ~ 11,000 genes (see the numbers at the bottom (15 M, etc) for the estimated number of reads required for each sample type, and see methods for more details). (**d**) The average coverage along the relative genomic region from the 5′ end (Transcription Start Site) to the 3′ end (Transcription End Site) for each sample. mRNA-seq protocol at the left (purple; solid line for FF samples (T1-T3) and dashed line for FFPE samples (T1-T6)). RiboZero protocol to the right (orange; solid line for FF samples (T1-T3) and dashed line for FFPE samples (T1-T3))

To accurately and sensitively estimate gene expression, high coverage of exonic regions is needed. Comparing the fractions of exonic reads out of uniquely mapped reads demonstrated that mRNA-seq was superior to RiboZero (Fig. 1a). While both protocols showed lower fraction of exonic regions for FFPE compared to FF, mRNA-seq resulted in a total of 42–61% exonic regions compared to 2–14% for RiboZero. Larger fractions of reads were mapped to intronic regions in the RiboZero libraries, and this significantly varied between samples.

To estimate the total number of reads one needs in order to get expression data for a satisfactory number of genes from each protocol, we compared the fraction of exonic reads out of total reads (Fig. 1b). Again, for all protocols the percentages of reads mapping to exons (out of the total number of reads) were lower in FFPE RNA libraries compared to FF (about 1/2). However, focusing on FFPE samples reveals that while the exonic fraction is around 30% for the mRNA-seq protocol, only about 10% of the total reads remain for analysis in the RiboZero protocol (Fig. 1b). This key point is further demonstrated when calculating the estimated total number of reads required to detect approximately 11,000 genes (threshold is set according to the saturation of the curves in Fig. 1c). While with mRNA-seq the total number of reads needed to reach this endpoint is 26–42 million for FFPE samples, depending on their quality and age (range between ~ 4 to ~ 10 years old), the RiboZero protocol requires at least 70 million reads for the ~ 4 years old samples (Fig. 1c).

As the mRNA-seq protocol captures transcripts based on their poly(A) tail, a 3′-end bias is expected, predominantly for degraded transcripts. To check the extent to which the library preparation protocol generates a 3′-bias, we plotted the coverage along the normalized transcript length (Fig. 1d). A small 3′-end bias is evident for FF samples in both protocols. In the RiboZero protocol, no difference was observed between FF and FFPE. For mRNA-seq libraries, the 3′-end bias is much more evident for FFPE samples, indicating that with this protocol fragmented RNA transcripts are mainly represented at their 3′-end.

### Total gene expression correlation

**Fig. 2:**
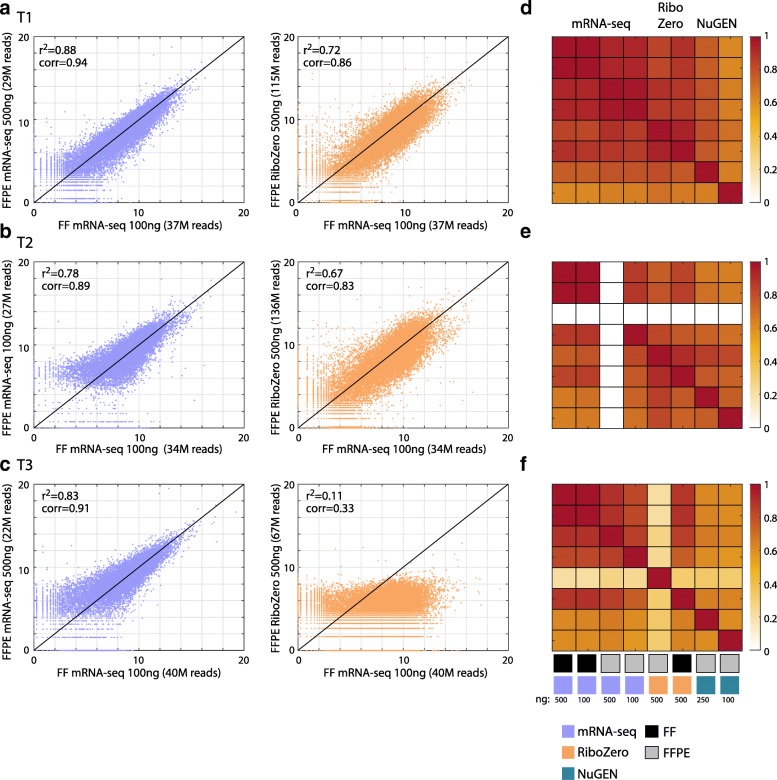
Comparisons of matched FF and FFPE samples prepared with different RNA-seq protocols. (**a**) Scatter plot of gene expression levels as measured from FFPE sample T1 by mRNA-seq protocol (purple; left) and RiboZero protocol (orange; right), compared to the gene expression data obtained from the FF sample of the same tumor . Gene expression data presented in log2 scale; r-square and correlation coefficients are presented for each plot. Total number of reads for each library is indicated at the x and y labels (M = million) (**b**) Same as (**a**) for the T2 samples (**c**) Same as (**a**) for T3. (**d**) Pearson correlation coefficient matrix between T1 gene expression data as measured by the different protocols: mRNA-seq in purple, RiboZero in orange and NuGEN ovation in blue, on the FF (black) and FFPE (gray) samples (colorbar at the bottom), measured for the indicated RNA quantities (in ng). The colorbar to the right is for the correlation coefficient values from 0 to 1. (**e**) Same as (**d**) for T2 (**f**) Same as (**d**) for T3

The correlation of gene expression between matched FF and FFPE samples for mRNA-seq and RiboZero protocols are shown in Fig. 2a-c. For all the three tumors, the mRNA-seq protocol resulted in high correlation between FF/FFPE pairs (correlation coefficient around 0.9) whereas the riboZero protocol yielded lower correlations for tumors T1 and T2 and no correlation for tumor T3, which failed exome mapping. A color-coded correlation matrix comparing all methods and the various initial RNA amounts is shown in Fig. 2d-f. mRNA-seq protocol resulted in highest correlation between matched FF and FFPE both for 500 and 100 ng. Overall, it appears that the mRNA-seq protocol provides consistent correlation between matched FF and FFPE total gene expression, even for samples that the RiboZero protocol failed to show with similar number of reads. These results suggest that FFPE samples can be a reliable alternative for FF samples by mRNA-seq also at low initial amounts of 100 ng FFPE-derived RNA.

### Differential gene expression

**Fig. 3:**
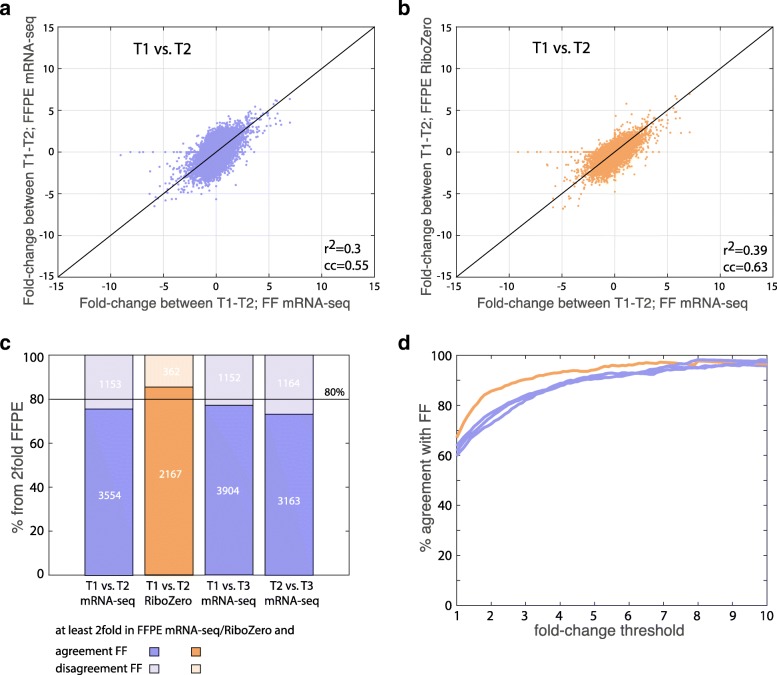
Comparison of fold-changes measured for FFPE samples versus matched FF samples. (**a**) Scatter plot for the expression fold changes (log2 scale) of ~ 23,000 genes measured in T1 vs. T2, obtained from FF samples (x-axis) compared to matched FFPE samples (y-axis) by mRNA-seq protocol (purple). R^2^ and correlation coefficient are presented at the plot. (**b**) Scatter plot for the expression fold changes (log2 scale) of ~ 23,000 genes measured in T1 vs. T2, obtained from FF samples (x-axis) compared to matched FFPE samples (y-axis) by RiboZero protocol (orange). R^2^ and correlation coefficient are presented at the plot. (**c**) For each comparison in (**a**)-(**b**) the percentages of differentiating genes by FFPE samples (2fold) that are in agreement or disagreement with the FC of matched FF samples are presented (see methods for more information). In addition, the same percentages are presented for the FC comparing T1 vs. T3 and T2 vs. T3 for the mRNA-seq protocol. Purple bars represent the mRNA-seq protocol and orange bar for RiboZero protocol. (**d**) Same as (**c**), presented for fold-change threshold values (imposed on the FFPE samples) varying between 1 to 10 (x-axis). The y-axis shows the percentage of differentiating genes in FFPE that changed in the same direction as in matched FF samples. Purple for mRNA-seq protocol and orange for RiboZero protocol

The ideal quality check for RNA-seq data is to evaluate its reliability to quantify differential gene expression. To estimate this, we compared the fold changes (FC) between two different FFPE samples to the FCs between their matched FF samples (Fig. 3a-b). Both mRNA-seq and RiboZero protocols yielded high correlation between the FCs of two FFPE tumor samples and the FCs obtained from their matched FF samples. Since the RiboZero protocol failed for tumor T3, we were able to compare only the differences between tumors T1 and T2, while for the mRNA-seq protocol we were able to compare all three comparisons between T1-T3 (Additional file 5: Figure S2). To evaluate the extent of agreement in differential expression between FFPE and FF samples, we calculated the fraction of genes with FC ≥ 2 between two FFPE samples, which change in the same direction in the matched FF samples (see methods for details). As demonstrated in Fig. 3c, the percentage of agreement between FFPE samples and matched FF samples is comparable between mRNA-seq and RiboZero protocols (around 80%), with a slight advantage for the latter. We further checked the dependence of this percentage of genes with FC in agreement on the value of the FC threshold used (between 1 to 10 fold, Fig. 3d), yielding similar results. Notably, in addition to the technical differences between the protocols, the observed variability between differential gene expressions of matched FF and FFPE samples can be due to expression noise and tumor heterogeneity, as the two sample types were taken from two regions of the tumor.

### Biological and clinical validity of gene expression

**Fig. 4:**
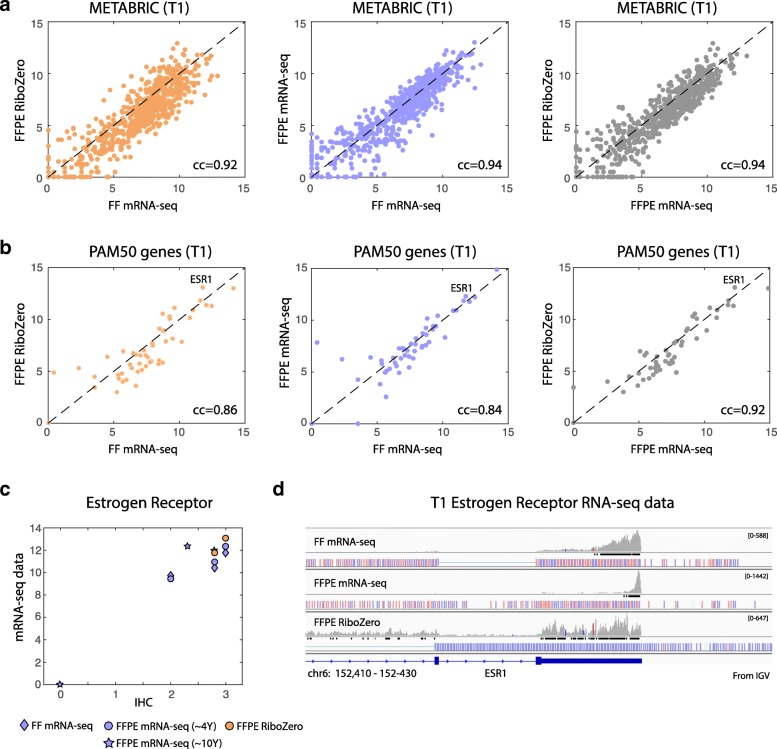
Expression of clinically relevant genes in breast cancer, and comparison to IHC. (**a**) Scatter plot of the expression levels of a set of 701 differentially expressed genes in the METABRIC dataset, between normal and tumor samples, as measured on T1 FFPE sample by RiboZero (orange; left) or mRNA-seq (purple; middle) vs. their mRNA-seq derived expression in the T1 FF sample. At the right we compare the expression of these genes on the T1 FFPE sample alone, as measured by mRNAs-seq (x-axis) and RiboZero (y-axis). (**b**) Same as (**a**) for PAM50 genes (“intrinsic subtype”). ESR1 (Estrogen Receptor) is indicated in the scatter plots. (**c**) Comparison between the expression levels of Estrogen Receptor (ESR1) as measured by the different RNA-seq protocols on T1-T6 (mRNA-seq in purple, RiboZero in orange, diamond for FF samples, circles for ~ 4 years old FFPE samples, and stars for ~ 10 years old FFPE samples), relative to IHC levels. (**d**) Chromosomal view of ESR1 with the reads mapped to this location (from IGV). Data shown for T1 FF sample, done with the mRNA-seq protocol; T1 FFPE sample, done with mRNA-seq protocol, and T1 FFPE sample done with RiboZero protocol. For each panel a histogram of the mapped reads to this genomic location is presented. At the bottom the gene model and the chromosomal location are shown

To estimate the biological and clinical significance of the results to breast cancer, we tested the correlation between the expression levels of the various protocols for relevant gene lists. First, we focused on 701 genes that are differentially expressed between normal breast and breast cancer, derived from the METABRIC dataset [2] (Fig. 4a). Comparing the standard FF mRNA-seq to the FFPE RiboZero, which is the comparison done so far in other studies, results in high correlation for these genes (cc = 0.92) (left, orange). Notably, a general shift of higher expression in the mRNA-seq is presented by higher fraction of genes below the diagonal (× 2.5 fold). Comparing expression levels between FF and FFPE samples, both with the mRNA-seq protocol (middle, purple) removes this shift (fractions of genes below and above diagonal are similar) and results in higher correlation levels (cc = 0.94). Here, the method is the same but the small variability may stem from expression noise of the two adjacent tumor regions. The ideal comparison between the two methods applies both to the same FFPE sample (right, grey) and results in high correlation (cc = 0.94). But similarly to the first comparison between the 2 methods, the mRNA-seq protocol results in higher expression levels versus the RiboZero, as shown by higher fraction of genes bellow the diagonal. This indicates that the higher expression levels are related to the mRNA-seq method rather than to the sample quality, as could be assumed on the basis of the first comparison, performed in previous studies. This is further emphasized in the comparison of the two sample types by the same mRNA-seq method, showing no advantage for the FF sample (similar amount of genes bellow and above diagonal; middle, purple). Notably, the high correlation between the methods is evident across all expression levels, except at the very low expressed genes below 5 (log2 scale). A further zoom-in into the correlation between the methods is presented by comparing the expression levels of the PAM50 genes [15] (Fig. 4b). At this resolution the high correlation between the expression levels of both methods for these clinically relevant genes is more noticeable.

Additional biological relevance of the expression data is demonstrated by comparing the immunohistochemistry score of estrogen receptor to its expression level (Fig. 4c). There was a very good agreement between the ESR1 expression level and its pathological score, even for the older FFPE samples from ~ 10 years ago (T4-T6; marked by stars in Fig. 4c).

Importantly, the correlation between the mRNA-seq and the RiboZero method is very high despite the difference in distribution of the reads across the genes. A representative plot of the reads obtained for each method is illustrated at the chromosomal map of the ESR1 gene (Fig. 4d). It is clear that the number of reads at the 3′-end of the transcript is much higher for the mRNA-seq protocol than the RiboZero protocol and this is much more evident in the FFPE sample. The RiboZero library is distributed across the entire exome as well as at intergenic regions. Nevertheless, as shown in Fig. 4a-c, expression levels are very similar despite the difference in distribution of reads across the gene.

## Discussion

Genome profiling datasets of many tumors types are the basis for a large number of studies, in both basic research and clinically relevant diagnostic developments. Most studies are performed on fresh frozen biopsies, assuming that the much more readily available FFPE samples are less suitable for large-scale genomic profiling. The availability of FFPE tumors at pathological archives enables researchers to expand the current expression databases to specific tumor subtypes, to perform extensive retrospective analyses and to execute longitudinal studies on individual patients over the course of disease. In this study, we demonstrate that standard mRNA-seq of FFPE samples is a consistent and efficient method for basic transcriptome profiling.

RNA-seq is increasingly gaining ground as the preferred unbiased method for genome profiling. Indeed, several previous studies have already showed high correlation between matched FF and FFPE samples, indicating feasibility of RNA-seq for the latter [10, 12–14, 16–18]. However, most studies compared the standard poly(A) mRNA-seq protocol for FF samples to ribosomal depletion, used for FFPE samples [10, 12–14]. Alternatively, FF samples were compared to matched FFPE using ribosomal depletion methods for both sample types [16–18] (Additional file 3: Table S3). When FF samples are compared to matched FFPE, using different protocols for the two sample types, it is difficult to interpret the results since difference can be caused by both factors – different samples and different protocols. Nevertheless, the “ideal” comparison, of results obtained for matched samples of the two types, using mRNA-seq for both, was not yet performed. The reason is that it was assumed that oligo(dT) primers would not reliably capture the partially degraded and chemically modified transcripts, and therefore gene expression will be underestimated in an unknown transcript-dependent manner. These studies concluded that RiboZero is a satisfactory method for RNA-seq of FFPE, compared to the gold standard mRNA-seq of matched FF. But the ribosomal-depletion methods frequently resulted in lower fraction of exonic transcripts out of total reads (10–24%, depending on the initial starting material, Additional file 3: Table S3), requiring significantly higher numbers of reads (55 M–65 M reads) ([10, 12–14, 16–19]). A large fraction of reads obtained by the ribosomal-depletion methods map to intronic regions and to unspliced mRNA [17].

Here we compared matched FF and FFPE samples using both the standard protocol for mRNA-seq (Truseq) and the RiboZero method, used for both tissue types. The results indicate that gene expression measurement of FFPE tissues using the standard poly(A) protocol is feasible and represents similar differential expression as obtained for FF tissues. Our results are consistent with a study by Beck et al., showing that their customized 3’end sequencing is an effective technique for expression profiling of FFPE samples [20].

Although we observed a 3′-end bias in the mRNA-seq libraries of FFPE compared to the RiboZero libraries, this bias was not reflected in the expression levels. Gene quantification was highly equivalent for a wide range of expression levels. Estimation of expression fold changes between FF and FFPE samples from both protocols was also very consistent, resulting in high overlap, in differentially expressed genes and their fold changes, between the two methods and sample types.

The main advantage of the mRNA-seq protocol over the RiboZero protocol is the higher percentage of exonic reads out of the total number of reads, which enables to quantify gene expression with much lower coverage, and hence it is a cost-effective approach. In addition, in the protocol we select the desired genomic regions (i.e. exons, mRNA), as opposed to the RiboZero protocol, in which we deplete some of the undesired regions (i.e. the rRNA). Thus, mRNA-seq requires less preliminary calibrations (e.g. calibration of the beads required for efficient depletion of rRNA in the RiboZero protocol).

Our results suggest that for standard transcriptome profiling, mRNA-seq of FFPE is a reliable and cost-effective method. With this said, it is important to keep in mind the limitations of the mRNA-seq methods. Since this method sequences mainly the 3’end of the transcript, it excludes transcripts that do not have poly(A) tails. Notably, for the non-coding lincRNAs that are mostly polyadenylated we observed a very good correlation between the RiboZero and mRNAseq libraries of the FFPE samples (Additional file 6: Figure S3A). Both methods, however, are not ideal for short non-coding RNAs, such as miRNAs, that require size selection steps, yet the very few detected miRNAs were similarly represented in the two methods (Additional file 6: Figure S3B).

In the case of highly degraded samples, mRNA-seq will not be ideal for accurate sequence-based discoveries such as identification of novel transcripts, alternative splicing or of single nucleotide polymorphisms. Low quality samples can be related to archival time as well as to preservation methods. All the FFPE samples in this study were of low quality, as measured by the RNA integrity number. For a relatively moderate time of archiving, starting material of 100 ng of RNA is sufficient and comparable to 500 ng of RNA. For highly degraded samples, such as very old archived samples, the minimal amount of RNA is 500 ng or higher.

## Conclusions

In summary, when considering methods for transcriptome profiling, practice and cost are important factors. Ribosomal depletion protocols have higher cost per sample and require 2–3 times more reads to compensate for higher fraction of intergenic reads. mRNA-seq method has lower library as well as sequencing costs due to higher fraction of exonic reads. Our results demonstrate that conventional transcriptome profiling of FFPE tissues is feasible as an alternative for frozen samples. Although mRNA-seq libraries are mainly represented by the 3′-end relative to the uniform distribution of reads by the RiboZero method, the resulting gene expression levels are highly comparable and obtained at much lower total numbers of reads.

## Methods

### Tumor samples

FF breast cancer tumor samples, collected for the Sheba Medical Center institutional tumor bank at time of surgery were included (*n* = 3). Matched FFPE blocks were obtained from the same resected tumors, archived at the Sheba Pathology Institute (preservation time of 4–5 years). All patients had estrogen receptor positive tumors. Three additional FFPE tumor samples, with longer preservation times (~ 10 years), were included. Tissue slides were examined by expert breast pathologists to include a minimum of 70% cancer cells. The study was approved by the Institutional Review Board (IRB).

### RNA extraction

FFPE tumor samples were sectioned to 5 μm slices and deparafinized at 90 °C for 5 min. Total RNA was extracted using nucleic acid isolation kit (AllPrep® Qiagen) according to the protocol instructions, with the following modifications: DNAse treatment was at 37° for 20 min and the column was incubated for 1 min before each washing step with buffer RPE. Samples were eluted in 30 μL purified water. RNA from FF tumor samples was extracted using Trizol. RNA concentrations were determined by Qubit™ fluorometer (Thermofisher scientific) and the RNA quality was determined by measuring the RNA integrity number (RIN) using Agilent TapeStation.

### Library preparation

Library preparation and sequencing were performed at the Nancy and Stephen Grand Israel National Center for Personalized Medicine at the Weizmann Institute of Science. Total RNA from FFPE and FF samples was processed using three protocols:

1. Truseq RNA Sample preparation kit v2 (illumina) (cat# RS-122-2002) for mRNA-seq libraries. Briefly, polyA fraction (mRNA) was purified from 500 ng or 100 ng of total RNA following by fragmentation and generation of double stranded cDNA. Then, end repair, A base addition, adapter ligation and PCR amplification steps were performed.
2. Truseq Stranded total RNA with Ribo-Zero Gold. Library Protocol: TruSeq® Stranded Total RNA (Illumina) (Cat # RS-122-2301, RS-122-2302) for ribosomal-depletion libraries. Briefly, After rRNA depletion from 500 ng total RNA with rRNA Ribo-Zero Gold removal mix, cDNA was performed followed by second strand synthesis with dUTP instead of dTTP. Then, A base addition, adapter ligation, UDG treatment and PCR amplification steps were performed.
3. Ovation Human FFPE RNA-Seq Library (Cat # 0340–32, NuGEN) for ribosomal-depletion libraries. Starting material for the libraries was: 100 ng for fresh frozen samples whereas 100 and 250 ng for FFPE samples.

Libraries concentrations were evaluated by Qubit (Thermofisher scientific) and their size was evaluated by Agilent Tapestation. Primer dimers were eliminated using 1× Agencourt RNAClean XP Beads after library preparation. Sequencing libraries were constructed with TruSeq SBS Kit using barcodes to allow multiplexing of few samples in one lane with a read length of 60 bp single-end run in Illumina HiSeq V4 instrument.

### Immunohistochemisty

Diagnostic slides were immunostained for Estrogen receptor (ER) on the Ventana Discovery autostainer (Ventana) using commercial ER antibody. ER levels were determined by a dedicated breast pathologist in accordance with the clinical guidelines by the American Society of Clinical Oncology (ASCO) and the College of American Pathology (CAP).

### Data analysis

#### Processing and alignment

Fragments were mapped to the human genome (hg19) using TopHat version V2.0.5 [21]. Only uniquely mapped fragments to the genome were considered and exonic and intronic signals were calculated using HTSeq [22]. The signals of the same sample from all lanes were summed. The number of fragments obtained for each gene in each sample was normalized to the total number of fragments obtained from this sample, and comparisons were made between the expression levels of a gene across all samples and not between different genes. The minimum expression level threshold was set to 5 (log2 scale) to reduce noise (based on data distribution).

#### Coverage along the genomic region

The average coverage along the genomic region from 5′ transcription start site to 3′ transcription end site was calculated for each sample using ngs.plot [23].

#### Estimation of total number of reads required for each protocol and sample type

The number of uniquely mapped reads to exonic regions varied significantly between samples and protocols (from 1 to 20 million). For each library (total of 29 libraries from the different sample types and protocols), we counted the number of exonic reads and the number of detected genes; the resulting 29 points lied, to a good approximation, on a smooth monotonic curve (Additional file 4: Figure S1). For each protocol and sample type we estimated the percentage of exonic reads out of the total number of reads. Using the curve and this percentage, we extrapolated, for each protocol and sample type, the corresponding curve of the number of detected genes for a given total number of reads (see Fig. 1c).

#### Fold change agreement between FF and FFPE samples

We estimated the level of agreement between the differential expression (Fold Change, FC) of a gene, as measured in two FFPE samples, and the FC obtained from their matched FF samples. This was done for all genes with FC of at least 2 in the two FFPE samples (up- or down-regulated), to calculate how many of these genes exhibited FC in the same direction in the matched FF samples. To control the dependence of this measure of the FC agreement, the same procedure was repeated for FC thresholds varying between 1 to 10.

#### METABRIC dataset

The METABRIC dataset [2] contains mRNA expression data for ~ 2000 breast tumors and 126 normal breast samples. Two-samples t-test was performed on all expressed genes to compare their expression levels in normal versus tumor samples. Genes with 0.1% FDR were defined as differentially expressed between normal and tumor samples.

## Additional files

Additional file 1: **Table S1.** RNA integrity number for all samples. (PDF 212 kb)

Additional file 2: **Table S2.** Number of exonic and intronic reads. (PDF 167 kb)

Additional file 3: **Table S3.** Comparison of the mean % of reads mapped on exons in other studies. (PDF 147 kb)

Additional file 4: **Figure S1.** Number of detected genes as a function of exonic reads. The number of detected genes as a function of exonic reads is shown for each library (total of 29 libraries from the different sample types and protocols, see legend). The black line is a smooth monotonic curve extrapolated from all 29 data points. (PDF 722 kb)

Additional file 5: **Figure S2.** Comparison of fold-changes measured for FFPE samples vs. matched FF samples using mRNA-seq. (A) Scatter plot for the expression fold changes (log2 scale) of genes measured in T1 vs.T3, obtained from FF samples (x-axis) compared to matched FFPE samples (y-axis) by mRNA-seq protocol (purple). r-square and correlation coefficient are presented at the plot. B) Scatter plot for the expression fold changes (log2 scale) of genes measured in T2 vs.T3, obtained from FF samples (x-axis) compared to matched FFPE samples (y-axis) by mRNA-seq protocol (purple). r-square and correlation coefficient are presented at the plot. (PDF 936 kb)

Additional file 6: **Figure S3.** Expression of non-coding RNAs in FFPE samples by mRNAseq and RiboZero protocols. (A) Scatter plot of the expression levels of annotated lincRNAs as measured on T1 FFPE sample by mRNAseq (x-axis) versus RiboZero protocol (y-axis). Correlation coefficients between the two protocols for the expression of these lincRNAs are indicated at the fig. (B) Same as (A) for miRNAs expression. (PDF 185 kb)
