# Supplementary material for: mRNA-seq whole transcriptome profiling of fresh frozen versus archived fixed tissues
Source: BMC Genomics. 2018 May 30;19:419. doi: 10.1186/s12864-018-4761-3 (PMC5977534; doi:10.1186/s12864-018-4761-3)
Supplement: Supplementary file 1 — Table S1. RNA integrity number for all samples. (PDF 212 kb) [file 12864_2018_4761_MOESM1_ESM.pdf]

Table S1: RNA integrity number for all samples

Tumor	Frozen sample	FFPE sample
**T1**	7.0	Bellow detection limit
**T2**	6.7	1.8
**T3**	7.4	Bellow detection limit
