# Supplementary material for: mRNA-seq whole transcriptome profiling of fresh frozen versus archived fixed tissues
Source: BMC Genomics. 2018 May 30;19:419. doi: 10.1186/s12864-018-4761-3 (PMC5977534; doi:10.1186/s12864-018-4761-3)
Supplement: Supplementary file 2 — Table S2. Number of exonic and intronic reads. (PDF 167 kb) [file 12864_2018_4761_MOESM2_ESM.pdf]

mean # reads	mRNA-seq			RiboZero		Nugen	
	FF	FFPE	FFPE old	FF	FFPE	FF	FFPE
	n=6	n=5	n=3	n=3	n=3	n=3	n=6
exons	15,682,261	8,163,961	3,323,204	4,321,386	12,117,409	2,435,552	360,440
int/intergenic	6,805,218	7,663,591	4,143,807	9,000,027	90,444,840	7,735,736	3,373,633
