# Supplementary material for: mRNA-seq whole transcriptome profiling of fresh frozen versus archived fixed tissues
Source: BMC Genomics. 2018 May 30;19:419. doi: 10.1186/s12864-018-4761-3 (PMC5977534; doi:10.1186/s12864-018-4761-3)
Supplement: Supplementary file 3 — Table S3. Comparison of the mean % of reads mapped on exons in other studies. (PDF 147 kb) [file 12864_2018_4761_MOESM3_ESM.pdf]

Table S3

Reference	mean % mapped on exons out of total # reads *				RNA amount (ug)
	poly (A) FF	poly (A) FFPE	Ribo. Dep. FF	Ribo. Dep. FFPE	
Our data	58 %	29%	21%	8%	0.1 - 0.5
Sinicropi et al.				13%	0.1
Graw et al.	35%			10%	0.5
Hedegaard et al.			30%	12%	0.5
Esteve-Codina et al.			51%	24%	0.5-1
Zhao et al.	62%		31%	17%	1
Morlan et al.	54%		22%	21%	1
Adiconis et al.	58%			22%	1
Li et al.			35%	24%	4
Beck et al.	22%	14%			10

*Mean percentage of exonic reads out of the total number of reads was extracted from the data provided for each study by calculating the % of exonic reads out of the uniquely mapped reads.
