# Supplementary figures and images for: mRNA-seq whole transcriptome profiling of fresh frozen versus archived fixed tissues
Source: BMC Genomics. 2018 May 30;19:419. doi: 10.1186/s12864-018-4761-3 (PMC5977534; doi:10.1186/s12864-018-4761-3)

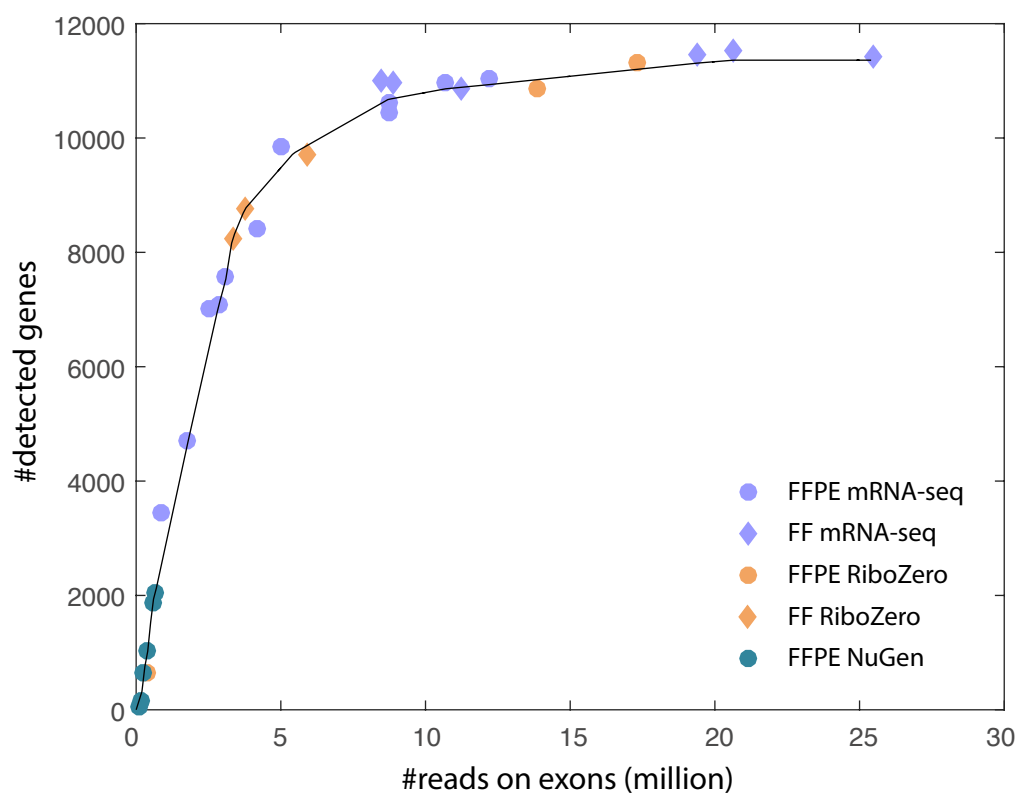

Supplement: Supplementary file 4 — Figure S1. Number of detected genes as a function of exonic reads. The number of detected genes as a function of exonic reads is shown for each library (total of 29 libraries from the different sample types and protocols, see legend). The black line is a smooth monotonic curve extrapolated from all 29 data points. (PDF 722 kb) [file 12864_2018_4761_MOESM4_ESM.pdf]

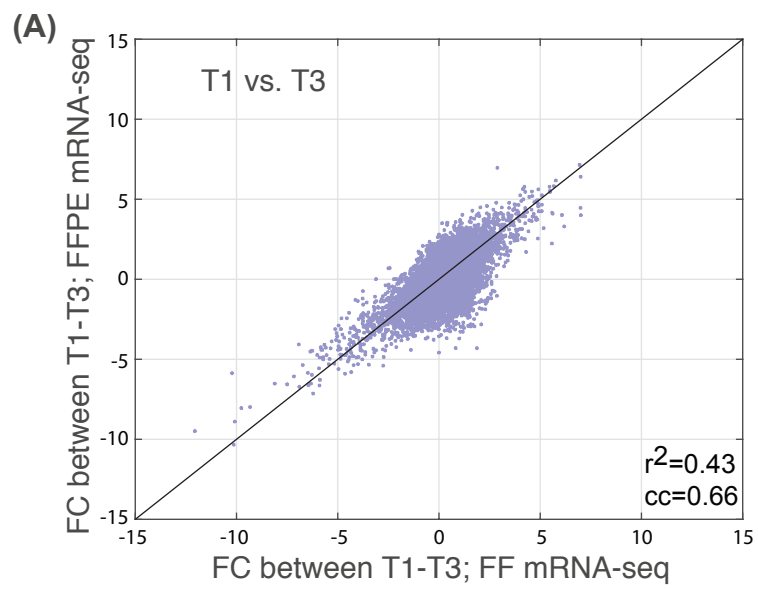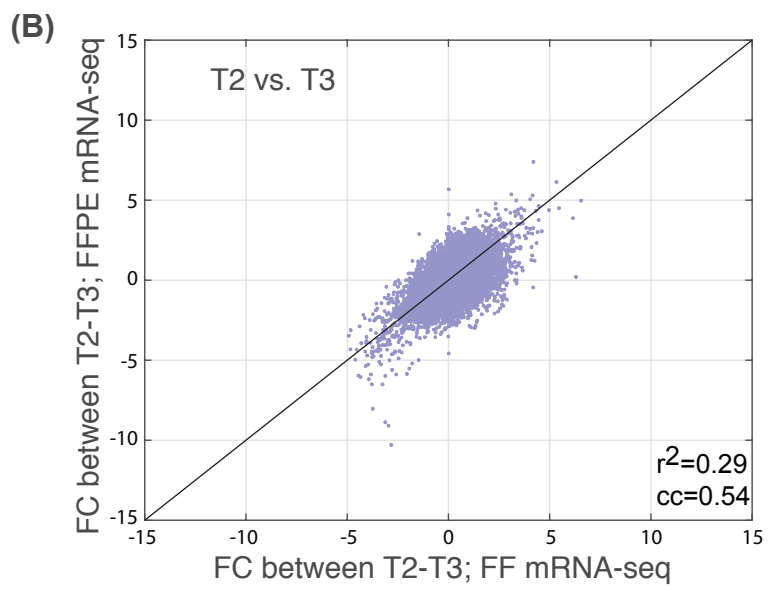

Supplement: Supplementary file 5 — Figure S2. Comparison of fold-changes measured for FFPE samples vs. matched FF samples using mRNA-seq. (A) Scatter plot for the expression fold changes (log2 scale) of genes measured in T1 vs.T3, obtained from FF samples (x-axis) compared to matched FFPE samples (y-axis) by mRNA-seq protocol (purple). r-square and correlation coefficient are presented at the plot. B) Scatter plot for the expression fold changes (log2 scale) of genes measured in T2 vs.T3, obtained from FF samples (x-axis) compared to matched FFPE samples (y-axis) by mRNA-seq protocol (purple). r-square and correlation coefficient are presented at the plot. (PDF 936 kb) [file 12864_2018_4761_MOESM5_ESM.pdf]

**A****LincRNAs (T1)**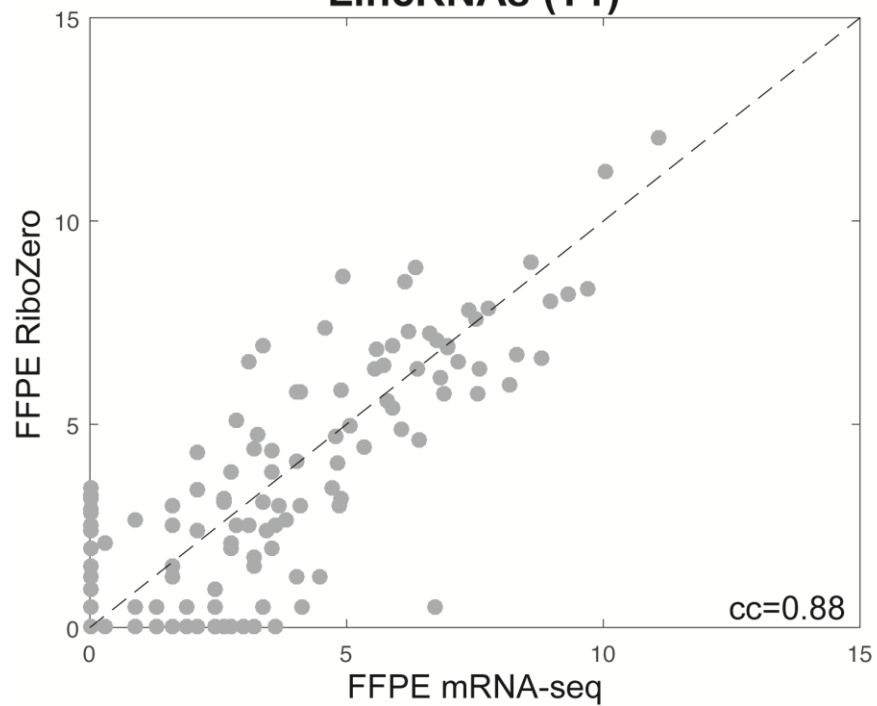**B****miRNAs (T1)**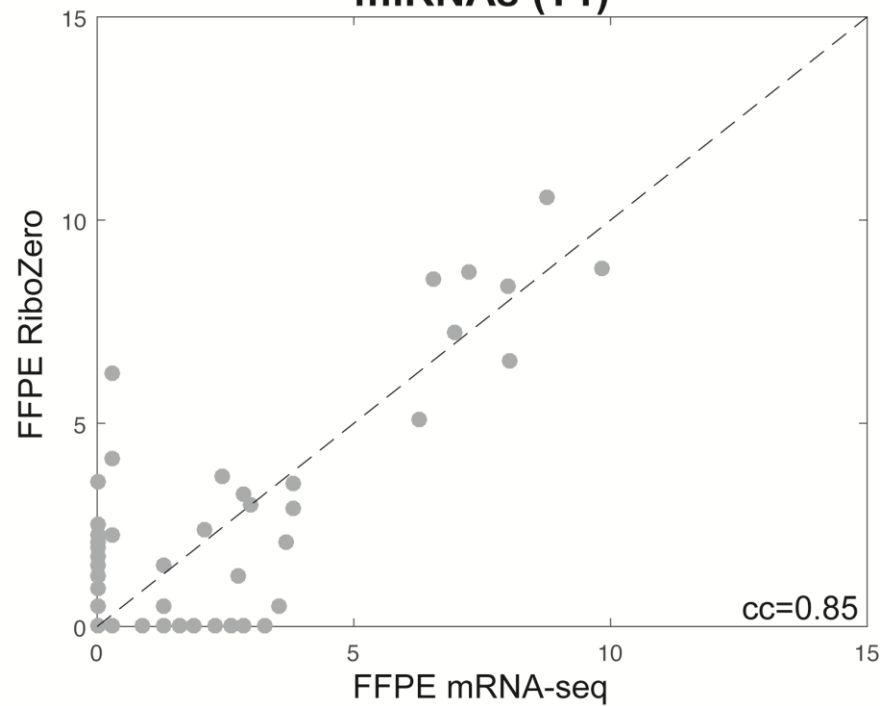

Supplement: Supplementary file 6 — Figure S3. Expression of non-coding RNAs in FFPE samples by mRNAseq and RiboZero protocols. (A) Scatter plot of the expression levels of annotated lincRNAs as measured on T1 FFPE sample by mRNAseq (x-axis) versus RiboZero protocol (y-axis). Correlation coefficients between the two protocols for the expression of these lincRNAs are indicated at the fig. (B) Same as (A) for miRNAs expression. (PDF 185 kb) [file 12864_2018_4761_MOESM6_ESM.pdf]
